# Negative Self-Assessment of Health in Women: Association with Sociodemographic Characteristics, Physical Inactivity and Multimorbidity

**DOI:** 10.3390/ijerph19052666

**Published:** 2022-02-25

**Authors:** Thays Angélica de Pinho Santos, Rafael Alves Guimarães, Valéria Pagotto, Natália Del’ Angelo Aredes, Isabela Silva Levindo de Siqueira, Suiany Dias Rocha, Clarissa Irineu de Sousa Carrijo, Claci Fátima Weirich Rosso

**Affiliations:** 1 Federal Institute of Education, Science and Technology of Goiás, Campus Goiânia Oeste, Goiânia 74055-110, Goiás, Brazil; thayssantos.ifg@gmail.com; 2 Faculty of Nursing, Federal University of Goiás, Goiânia 74605-080, Goiás, Brazil; valeriapagotto@ufg.br (V.P.); naredes@ufg.br (N.D.A.A.); clarissa.carrijo@ufg.br (C.I.d.S.C.); claci@ufg.br (C.F.W.R.); 3 School of Social and Health Sciences, Pontifical Catholic University of Goiás, Goiânia 74605-010, Goiás, Brazil; isabelalevindo@gmail.com; 4 Municipal Department of Education, Goiânia 74610-155, Goiás, Brazil; suianydias@gmail.com

**Keywords:** health self-assessment, chronic disease, multimorbidity, women’s health, morbidity, primary care, healthy behavior

## Abstract

Introduction: Women present a higher prevalence of negative self-assessment of health (NSAH) when compared to men. However, there is a gap in the literature of factors associated with NSAH in women from developing countries such as Brazil. In addition, few studies have assessed the magnitude of the association between multimorbidity and NSAH in this population. Thus, the aim of this study was to evaluate the association between NSAH and sociodemographic characteristics, lifestyle and multimorbidity in women from the Midwest region of Brazil. Methods: A study based on data from the National Health Survey, a household survey that investigated health situation, lifestyle and risk factors for chronic diseases in the adult population of Brazil, was held. Sampling was performed in multiple stages. The selected women answered a standardized questionnaire on sociodemographic data, self-assessment of health and potential determinants. Poisson regression was used to analyze the association between NSAH and sociodemographic characteristics, lifestyle and multimorbidity. A significance level of 0.05% was established. Results: The study included 4233 women. The prevalence of NSAH found was 6.0% (95% Confidence Interval [95% CI]: 5.1–7.0%). There was an association between NSAH and advancing age, low schooling, physical inactivity and multimorbidity. Furthermore, there was an association between NSAH and diseases/disorders such as chronic back pain, systemic arterial hypertension, mental disorders, depression, cardiovascular diseases, stroke, cancer, hypercholesterolemia and diabetes mellitus. Conclusion: The prevalence of NSAH was low. A strong association was found between this variable and multimorbidity. In addition, increased age, low schooling and physical inactivity were predictors of NSAH in women.

## 1. Introduction

Chronic noncommunicable diseases (NCDs) have a significant impact on public health, resulting in high morbidity and mortality, and high costs for health services and society in general [1,2,3]. According to the World Health Organization (WHO), the mortality rate related to NCDs has increased globally, regardless of socioeconomic factors [1]. In 2016, NCDs accounted for about 71% of deaths worldwide, most of them in low- and middle- to low-income countries [1]. Approximately 55 million deaths are expected to occur in 2030 due to complications of NCDs [4].

In Brazil, NCDs also have a high burden, being one of the major causes of premature death in adults [1,5]. It is estimated that NCDs represent 72% of the causes of death and that the probability of premature death in the country is 17% [1]. In 2016, the Global Burden of Disease (GBD) report estimated that there were 314,438 deaths from NCDs in individuals between 30 and 69 years of age, resulting in a standardized mortality rate of 340.4 deaths per 100,000 inhabitants [6].

The high burden of NCDs is due to the aging of the population and, above all, the increase in modifiable risk behaviors, such as smoking, alcohol abuse, physical inactivity, and inadequate diet, together with important social determinants in health [7]. These factors have an important impact on the reduction of functionality and are also related to the low quality of life of the population [8]. They also burden health services by increasing demand and are responsible for a large part of the countries’ Gross Domestic Product (GDP) through direct and indirect costs [2].

The negative effects of NCDs are enhanced in the presence of multimorbidity. This condition is defined as the coexistence of two or more NCDs in the individual [9]. Accordingly, multimorbidity due to NCDs is increasing in low- and middle-income countries, such as Brazil, and challenging health systems [10]. Individuals with multimorbidity present a higher risk of premature mortality, decreased functioning of physical and mental health, reduced quality of life, and have an increased demand of health services [10,11,12].

Several studies have shown that multimorbidity is strongly associated with negative self-assessment of health (NSAH) [13,14,15,16]. This indicator is used to verify the assessment or perception that individuals have of themselves regarding their health, being essential for monitoring the health conditions of populations [17,18]. It is a multidimensional indicator that takes care of objective and subjective aspects, covering nuances related to social health constraints and determinants [18]. It is also known that NSAH is associated with the presence of NCDs, morbidity, mortality and is also related to the presence of risk factors as previously described in the context of NCDs [17,18,19,20]. The literature found sustain that there is an association between sedentary lifestyle and NSAH [17], reporting that 87% of women analyzed in this specific research were physically inactive. Additionally, an association between NSAH and higher index body mass (IBM) is recognized, similarly to its association with female gender [21], pointing to behavioral aspects related to women’s lifestyle that must be addressed to avoid NSAH and NCDs.

Health self-assessment has a global evaluative nature, which considers several aspects of health, both subjective and objective, combined within the perceptive structure of the individual. Due to its ability to predict mortality and morbidity, health self-assessment is as an important indicator of the health status of populations. Self-assessment of health has a multidimensional structure, which comprises socioeconomic status, social support, physical health conditions, and access and use of health services, which are relevant to characterize people’s health status. Thus, self-rated health may reflect the presence of multimorbidity, which increases complex and continuous demands for care, greater use of health services and eventually complications that acutely affect health. Studies shows that poorer self-rated health is a reflection of the higher burden of disease, such as hypertension [22], smoking and leisure-time physical inactivity [23], stroke, musculoskeletal and other pain disorders, and three or more diseases [24].

Women tend to have a higher prevalence of NSAH compared to men [25]. In general, Brazilian women also presented a worse landscape of NSAH compared to men [18,26]. The incidence of NSAH in one study conducted with 1339 participants was 24.0 in men and 30.0% in women [26]. A larger investigation with 59,758 participants found that 6.8% of women reported NSAH compared to 4.8% among men, and male participants presented better positive self-assessment of their health, except when compared to women with more financial resources, privileged social groups and non-participant of the workforce were considered as variables in this analysis [18]. This is caused especially by gender inequalities, in addition to the higher prevalence of physical and mental NCDs and multimorbidity in women [18,25,27]. However, there is a gap in the literature in developing countries [25], such as Brazil, on the factors associated with NSAH in women and, especially, surveys on its association with multimorbidity. Most available studies conducted analyses grouped between genders or investigated associated factors in specific subgroups (e.g., elderly), using non-representative samples and did not assess the impact of multimorbidity on NSAH.

Thus, it is essential to investigate the factors associated with NSAH in women, especially multimorbidity, so that developing countries can develop strategies and public policies for the integral care of this population, aiming at surveillance of NCDs, reduction of the magnitude of NCDs, prevention measures and increased quality of life. The aim of this study was to analyze the association between NSAH and sociodemographic characteristics, lifestyle and multimorbidity in women from the Midwest region of Brazil.

## 2. Materials and Methods

### 2.1. Data Source

This was a cross-sectional, population-based study, using the National Health Survey (Pesquisa Nacional de Saúde—PNS) conducted in 2013 as a data source. The PNS is a survey conducted by the Ministry of Health in partnership with the Oswaldo Cruz Foundation and the Brazilian Institute of Geography and Statistics (IBGE). The general objective of this survey is to characterize the health conditions of the Brazilian population, lifestyle, access and use of health services, among other aspects [28,29].

The population of the NHS was represented by adult individuals aged 18 years or older living in all regions of Brazil. The previous study presented the data in a way grouped by sex and country [19]. In the present study, we analyzed data from women living in the four states of the Midwest region of Brazil: Mato Grosso, Mato Grosso do Sul, Goiás and the Federal District.

The sampling was done by clusters in three stages. The first was composed of the Primary Sampling Units and included census tracts of the selected municipalities. The second included the Secondary Sampling Units, which included permanent private households, defined as those intended to serve as housing for one or more people. Finally, the third stage included the Tertiary Sampling Units, composed of the resident of the selected household who answered the interview. A resident person was defined as the one who remained at home for at least three days a week. In all stages, the units were selected by simple random sampling [28,29]. 

Data collection was performed by trained professionals using Personal Digital Assistants. The questionnaire used in the PNS was validated by specialists and included collection of multiple variables. In the present study, variables with data on sociodemographic characteristics, lifestyle and presence of self-reported NCDs were used [28,29].

### 2.2. Variables

#### 2.2.1. Dependent Variable

The dependent variable was NSAH, categorized dichotomously as “0—No” or “1—Yes”. The primary variable used for its construction was obtained from the question “In general, how do you evaluate your health?”. The response options were organized on a Likert scale: “very good”, “good”, “fair”, “poor” or “very poor”. In this study, the participant who reported poor or very poor self-rated health was considered with NSAH [30].

#### 2.2.2. Independent Variables

The following independent variables were taken into account:

(i) Sociodemographic

Age group, stratified in the following groups: 18–39 years, 40–59 years and >60 years [31]; schooling (without schooling/incomplete elementary school, complete elementary school/incomplete high school, complete high school/incomplete higher education or complete higher education or higher) [19]; self-reported race/skin color, categorized as white, black, brown or others that included the indigenous and yellow categories due to the small number of observations in the sample [32]; marital status (with spouse or without spouse); residence area (rural or urban) and; state of residence (Goiás, Mato Grosso, Mato Grosso do Sul or Distrito Federal).

(ii) Lifestyle

(ii.1) Recommended consumption of vegetables and fruits (no or yes), defined as the daily consumption of at least 400 g of fruits and vegetables, which is equivalent to five portions or more a day, on five or more days of the week [33]. Consumption was measured based on questions about the frequency of ingesting raw and/or cooked vegetables and fruits: “On how many days of the week, do you usually eat lettuce and tomato salad or any other vegetable or raw vegetable?”; “In general, how many times a day do you eat this type of salad? (If Yes, in the previous answer) ”; “On how many days of the week do you usually eat vegetables or cooked vegetables, such as cabbage, carrots, chayote, eggplant, zucchini? (Not counting potatoes, cassava or yams)”; “In general, how many times a day do you eat vegetables or cooked vegetables? (If Yes, in the previous answer) ”; “On how many days of the week do you usually drink fresh fruit juice?”; “In general, how many glasses a day do you drink of fresh fruit juice? (If yes, in the previous answer) ”; “How many days a week do you usually eat fruit?” and “On how many days of the week do you usually eat fruit? (If yes, in the previous answer.” The sum of the amount of daily consumption was performed to compute the variable on a continuous basis, being considered regular consumption when the sum was equal to or greater than five.

(ii.2) Consumption of meat and/or chicken with excess fat (no or yes), individuals who reported consuming chicken with skin or meat with fat regardless of the amount consumed and weekly frequency [34]. Consumption was assessed based on the questions: “When you eat red meat, do you usually: (1. Remove the visible excess fat/2. Eat with fat)?”, and “When you eat chicken, do you usually: (1. Remove the skin/2. Eat with the skin)?”.

(ii.3) Regular consumption of sweets (no or yes). Those who reported consuming sweets on five or more days of the week were taken into account [34]. This was obtained from the question: “On how many days of the week do you eat sweet foods, such as slices of cake or tart, sweets, chocolates, candies, cookies or sweet cookies?”

(ii.4) Regular replacement of meals with snacks (no or yes), defined when the participant reported replacing meals with snacks five or more days a week [35]. This was measured by the question: “On how many days of the week do you replace your lunch or dinner with sandwiches, snacks or pizzas?”

(ii.5) Regular consumption of soft drinks and/or artificial juice (no or yes), which corresponds to the intake of soft drinks or artificial juices on five or more days per week [35]. Obtained from the question: “On how many days a week do you usually drink soda (or artificial juice)?”

(ii.6) Regular consumption of alcoholic beverages (no or yes), defined as the consumption of at least one dose of alcoholic beverage once or more per month [36]. Variable obtained from the questions: “How often do you usually consume an alcoholic beverage? (1-I never drink; 2-Less than once a month and 3-Once or more a month) ”.

(ii.7) Abusive alcohol consumption (no or yes), defined as the ingestion of up to four doses on a single occasion in the last 30 days [37]. This variable was obtained from the question: “In the past 30 days, did you consume 4 or more doses of alcoholic drink on a single occasion?”.

(ii.8) Smoking, categorized as non-smoker, ex-smoker and current smoker [31]. The questions used to construct this variable were: “Do you currently smoke any tobacco products? (1-Yes, daily; 2-Yes, less than daily and 3-I do not currently smoke) ”; “And in the past, did you smoke any tobacco products? (1-Yes and 2-No) ”- for those who answered no in the previous item.

(ii.9) Physical inactivity (no or yes). For this variable, the answers to questions related to physical activity in the last three months, frequency and duration of activity in the domains: Leisure physical activity, commuting physical activity and physical activity at work were taken into account. Physical activity scores were built in each of these domains by multiplying the weekly frequency by the time dedicated to activity performed on each day. The model questionnaire of the Surveillance System for Risk and Protective Factors for Chronic Diseases by Telephone Survey (Vigitel) [38] was used, which assesses the practice of physical activity in these three domains, using questions already addressed in international questionnaires used in the area such as the International Physical Activity Questionnaire. Women who stated practicing 150 min per week were considered physically active during leisure considering the sum of minutes of the three domains mentioned [39].

(iii) Multimorbidity

Defined as the presence of two or more chronic conditions, categorized as no or yes [9]. The following NCDs were considered for the computation of the variable: systemic arterial hypertension, diabetes mellitus, hypercholesterolemia, cardiovascular disease, stroke, asthma, arthritis/rheumatism, chronic back pain, work-related musculoskeletal disorder (WMSD), depression, mental disorder (schizophrenia, bipolar disorder, psychosis, obsessive compulsive disorder (OCD)), lung diseases (chronic obstructive pulmonary disease (COPD), pulmonary emphysema, chronic bronchitis), cancer, and chronic kidney failure. The question used for each NCD was: “Has any doctor ever given you a diagnosis of ______________?”.

### 2.3. Statistical Analysis

The data were analyzed using the STATA software (StataCorp LLC, version 16.0, College Station, TX, USA). Initially, a descriptive analysis of the variables assessed was performed. The characteristics of the sample were described as absolute (n) and relative (%) frequencies. Then, bivariate and multiple Poisson analyses were performed to investigate the magnitude of the association between the independent variables and the dependent variable [40]. Poisson’s bivariate analysis explored the association between each independent variable and the dependent variable; the results of this analysis were presented as the Crude Prevalence Ratio (PR) and respective 95% CI. Variables with *p* < 0.20 in this analysis were inserted in a Poisson multiple regression model to adjust for potential confounding variables. The final model was adjusted by age group, schooling, race/color, marital status, state of residence, smoking, alcohol abuse, recommended consumption of fruits and vegetables, regular consumption of soda and/or artificial juice, regular consumption of sweets, physical inactivity and multimorbidity. The results of this analysis were presented as an Adjusted Prevalence Ratio (aPR) and 95% CI. In addition to the general model, multiple regression models were performed to analyze the contribution of each NCD to the NSAH. The statistical significance of the Poisson models was verified by Wald’s statistics. Variables with *p* < 0.05 were considered statistically significant.

Poisson regression is generally used in epidemiology to analyze longitudinal studies where the response is the number of episodes of an event that occurred in each period. The model equation is:

$$\log\left(\frac{n}{t}\right)=\beta 0+\beta 1X1+\cdots +BkXk$$

where *n* is the number of events for a given individual, *t* the study period and *Xk* the covariates. The parameters (βi) of the model are log of relative risks or prevalence ratio.

### 2.4. Ethical Aspects

The PNS was approved by the National Research Ethics Commission of the National Health Council, protocol 328.159/2013. Written consent was obtained from all participants.

## 3. Results

### 3.1. Sample Characteristics

A total of 4233 women living in the Midwest region of Brazil participating in the PNS 2013 were included in the study, 1385 in the state of Goiás, 1067 in the Federal District, 986 in Mato Grosso do Sul and 795 in Mato Grosso. 

Table 1 shows the descriptive analysis of the sample. Regarding the age group, 48.5% of the population was between 18 and 39 years old; 35.2% of the women had low schooling (illiterate/complete elementary school); 51.5% self-declare themselves with brown skin; 59.7% had a spouse and 92.0% live in an urban area. Regarding lifestyle, a prevalence of recommended consumption of fruits and vegetables of 57.6% was identified; consumption of meat and/or chicken with excess fat of 37.0%; regular consumption of sweets of 21.3%; regular consumption of soft drinks and/or artificial juice of 23.5%. Furthermore, 7.3% of the women reported regularly replacing meals with snacks. Regular consumption and alcohol abuse was found in 28.5% and 9.0% of women, respectively. The prevalence of current smoking was 10.3% and the prevalence of physical inactivity was 53.7%. Finally, there was a prevalence of multimorbidity of 28.5% in the sample.

### 3.2. Health Self-Assessment

Of the total number of women, 13.4% (95% CI = 12.1–14.9%), 54.9% (95% CI = 51.9–55.9%), 26.8% (95% CI = 25.0–28.7%), 4.9% (95% CI = 4.1–5.8%) and 0.9% (95% CI = 0.6–1.4%) rated their health as very good, good, fair, poor and very poor, respectively. Thus, considering the definition adopted in this study, the prevalence of NSAH (poor/very poor) in the sample was 6.0% (95% CI = 5.1–7.0%).

### 3.3. Factors Associated with Negative Health Self-Assessment

Poisson’s bivariate analysis revealed an association between NSAH and the following variables: age group, schooling, race/skin color, recommended consumption of fruits and vegetables, regular consumption of soda, regular consumption of sweets, physical inactivity and multimorbidity, as shown in Table 2.

Table 3 shows the final Poisson model of the factors associated with NSAH. Variables with a *p*-value < 0.20 were included in the final model in the bivariate analysis. The model showed that the prevalence of NSAH was 2.43-fold higher (aPR: 2.43; 95% CI = 1.07–5.52) in women aged 40–59 years and 2.52 (aPR: 2.52; 95% CI = 1.02–6.23) fold higher in those aged 60 years or over, when compared to younger women. The prevalence was also 4.85-fold higher (aPR: 4.85; 95% CI = 2.27–10.37) in women with the lowest level of schooling (illiterate/incomplete elementary school) when compared to those with the highest level (higher education). This prevalence was 1.55-fold higher (aPR: 1.55; 95% CI = 1.11–2.15) in women who presented inactivity when compared to active women. Finally, the prevalence of NSAH was 6.93-fold (aPR: 6.93; 95% CI = 3.47–13.85) higher in women with multimorbidity when compared to those without this characteristic. These results indicate that multimorbidity is the condition that most impacts on women’s NSAH. In addition, they suggest an increase in negative self-assessment of health with increasing age and association with low schooling and physical inactivity.

**Figure 1:**
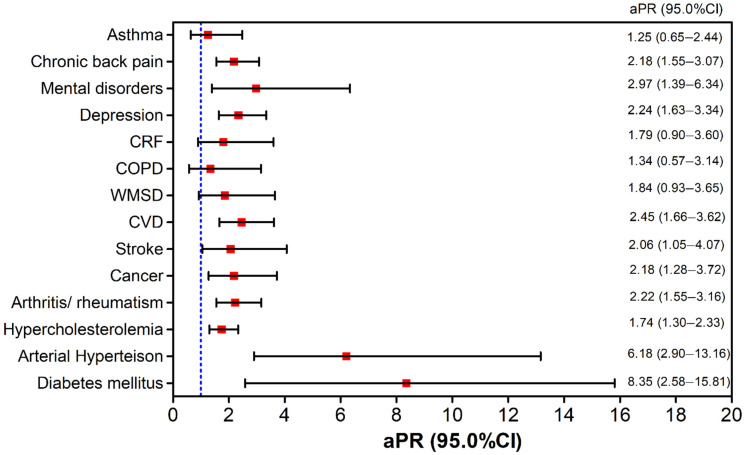
Analysis of the association between negative health self-assessment and chronic conditions in women in the Midwest region, Brazil, National Health Survey, 2013. aPR = Adjusted Prevalence Ratio; 95% CI = 95% Confidence Interval; WMSD = Work-related Musculoskeletal Disease; CRF = chronic renal failure; COPD = Chronic Obstructive Pulmonary Disease; CVD = cardiovascular diseases.

Figure 1 shows the aPR and 95% CI of the multiple regression models that assessed the magnitude of the association between each chronic condition and NSAH in women. There was a significant association between NSAH and chronic back pain, mental disorders (bipolarity, schizophrenia and obsessive compulsive disorder), depression, cardiovascular disease, cancer, stroke, arthritis, hypercholesterolemia, systemic arterial hypertension and diabetes mellitus.

## 4. Discussion

The results from this study revealed a low prevalence of NSAH in women in the Midwest region. Additionally, multiple regression analysis showed a positive gradient of NSAH with increasing age group; in addition to the association with low level of education and physical inactivity. Finally, a strong association between multimorbidity and NSAH was observed in the sample under study.

The prevalence of NSAH found in this study was 6.0% (95% CI: 5.1–7.0), similar to that found in the female population of Brazil in 2013 (6.8%) [18]. On the other hand, the estimated prevalence was statistically lower than that found in the female population of the country in 2008 (44.4%) [41] and 2003 (11.5%) [42]. Szwarcwald et al. [19] point out that this decrease is paradoxical, since the aging of the population and the increase in the magnitude of NCDs tend to increase the prevalence of NSAH, not decrease. However, increased access to health services, higher family income and education, in addition to the greater participation of women in the labor market over the years, may have contributed to the reduction in the prevalence of NSAH in recent years in Brazil [18]. The low prevalence found can also be attributed to the scope of the National Policy for Integral Attention to Women’s Health (PNAISM), which in Brazil has solid bases and actions in the field of well-established primary care, focusing on health promotion and disease prevention, such as NCDs [43]. 

This phenomenon demands a reflection on the dimensions involved in the NSAH construct. The association between NSAH and multimorbidity, found in this study, indicates that women possibly took into account the biological dimension of health in their perception. However, considering that this health indicator encompasses the physical, emotional components of well-being and satisfaction with one’s own life, it is hypothesized that: 1. in a gender perspective, it is possible that the low prevalence of NSAH may be the result of greater autonomy gained in different dimensions of life, active participation in different work spaces and conquest, although not entirely of equality between men and women; 2. that the health view is still restricted to biological aspects in common sense, disregarding or attributing less value to other factors contained in the holistic definition of health.

In the present study, a positive gradient was observed between the prevalence of NSAH and an increase in age group. In fact, studies have shown an increase in NSAH with increasing age in Brazilian women [18,19,44] and from other countries [23,45]. With increasing age, people are more susceptible to NCDs that compromise functionality and increase dependence, which can lead to an NSAH [17,18,23]. Studies on NSAH in the elderly show that the prevalence is higher than in other age groups, and the outcome of the increased prevalence of NCDs and unhealthy life habits throughout life [46,47]. Compared to men, this is an important result, because in old age, women still tend to undertake other roles in the family, such as caregivers of children, grandchildren, and elderly spouses, leading to overload, worse quality of life [48,49], and consequently NSAH. 

This study revealed an association between low level of education and NSAH in the study sample, indicating inequities of this indicator regarding the level of education. Similarly, other studies conducted with women have shown an association between education level and NSAH [18,45,50]. A survey that analyzed associations between educational level and NSAH among adults in Brazil from 1998 to 2013 showed a clear association between low schooling and NSAH, even with the improvement of schooling in the general population in recent years [50]. Evidence shows that education is a strong social determinant of health in Brazil, and individuals with lower education have lower income, higher prevalence of morbidity and lower access to health services, which contributes to a greater magnitude of NSAH at lower educational levels [18,27,50]. 

Social determinants and conditions, such as schooling, stand out in the analysis of the data of the present study, in the context of public health. These findings are not limited to Brazil, although the specificities of our sociocultural organization are important to analyze the findings and propose improvement strategies. For example, a study published in the USA identified higher NSAH among older and black women with low schooling and low income [51].

Physical inactivity was significantly associated with NSAH in the study sample, corroborating other studies conducted in women [16,17,23,52,53]. It is estimated that the prevalence of physical inactivity affects almost half of the Brazilian population (46.0%), with a higher frequency in women than men (51.5% versus 29.8%) [39]. The present study also showed a high prevalence of physical inactivity in women (53.3%). Studies indicate that insufficient physical inactivity is associated with several negative health effects, such as insulin resistance and type 2 diabetes; depressive symptoms, hypertension, obesity and other diseases that in turn influence NSAH [54]. Moreover, from a broader perspective of health, the relationship between regular physical activity and self-perception of health may be associated with better quality of life, general health and biopsychosocial well-being of women, self-care, independence, stress reduction and the risk of morbidity by various causes, including NCDs [55,56], which contributes to the positive effect of this variable on positive self-assessment of health. 

Finally, the present study showed a strong association between multimorbidity and NSAH, corroborating previous findings [16]. The prevalence of NSAH increased 6.93-fold in women with multimorbidity when compared to those without this characteristic. The strength of the associations between multimorbidity and NSAH differs between studies. A cross-sectional study conducted in Canada with women showed that the presence of two or more chronic health conditions increases the likelihood of NSAH by 9.73 fold [16]. Research conducted in England found that the likelihood of moderate or poor self-assessment increases 1.5 to 2.2 fold in the presence of two and three or more chronic conditions, respectively [13]. In Brazil, a study conducted in women using multimorbidity as a predictor of NSAH found that NSAH increased the probability of having two or more chronic conditions by 5.1-fold [57]. Studies that include mental disorders, such as depression, in the multimorbidity equation, display stronger likelihood of association, since these disorders are considered a major risk factor for NSAH [15]. 

The present study also showed that among the 13 diseases or groups of diseases investigated to estimate the prevalence of multimorbidity, 10 were statistically associated with NSAH after control of the other covariates. These diseases/health conditions included chronic back pain, systemic arterial hypertension, mental disorders, depression, cardiovascular diseases, stroke, cancer, hypercholesterolemia, and diabetes mellitus. However, the greatest strength of association was, in this order: diabetes mellitus, hypertension, mental disorders, depression and chronic back pain. All conditions require several procedures for their control, including changes in life habits, risk monitoring and drug treatment, which can imply complexity to the therapeutic regimen and influence the perception of one’s own health. Additionally, considering that self-perceived health is a multidetermined indicator, diseases such as diabetes and back problems bring long-term complications that may be tied to a dimension of physical health. Mental disorders and depression, in turn, may reflect women’s emotional health conditions [58,59].

### Limitations

This study has some limitations. First, the cross-sectional nature does not allow the establishment of cause-and-effect relationships between the dependent variable and the independent variables analyzed. Second, the data related to lifestyle were self-reported, susceptible to memory and response bias, and their magnitudes may be underestimated. To reduce memory and response bias, two strategies were used. To reduce memory bias, the validated questions used the shortest possible time periods (one week, one month or three months) or the habit of that behavior. To minimize response bias, the participants were interviewed by a researcher in a place of residence, without the presence of other family members. Third, NCDs measured to assess multimorbidity were also self-reported, and laboratory and/or clinical methods for diagnosis were not used and may also be underestimated. Fourth, the sample did not have the power to perform sensitivity analyses of the factors associated with NSAH, according to age groups, race/skin color and schooling, which are essential to assess differences in predictors according to sociodemographic characteristics. The small number of cases of the outcome (*n* = 248) did not allow the stratification of the sensitivity analyses, since it increased the 95% confidence intervals of the estimates and reduced the power of discrimination of the variables. Despite the limitations, this is one of the few studies that evaluated the magnitude of the factors associated with NSAH in a sample of women from Brazil, which may contribute to the formulation and implementation of public policies to improve the quality of life of this population.

## 5. Conclusions

In conclusion, the prevalence of NSAH found in this study was low (6.0%). Furthermore, NSAH was statistically associated with advancing age, low level of education, physical inactivity and multimorbidity. The findings of this study may contribute to the strengthening of public health promotion policies focused on primary health care aimed at improving the perception of women’s health through action on its determinants. This includes strengthening coping strategies for risk factors for NCDs, especially physical inactivity; actions to reduce NCDs, aiming at reducing the prevalence of multimorbidity; interventions aimed at more vulnerable groups, especially older women with low education. In Brazil, the Plan to Combat Chronic Noncommunicable Diseases 2021–2030 [60] is an example of a political initiative to face NCDs, which will impact the negative perception of health. Future studies should investigate NSAH as a predictor of morbidity and mortality in women; perform stratified analyses of the associated factors by age groups and analyze other variables associated with NSAH in this population.

## Figures and Tables

**Table 1:** Sociodemographic characteristics, lifestyle and multimorbidities in women. Midwest Region, Brazil, National Health Survey, 2013.

Variables	Total * (*n* = 4.233)	%	95% CI
Age group (years)			
18–39	1.013	48.5	45.2–52.0
40–59	1.478	35.0	33.1–36.9
≥60	743	16.5	15.0–18.1
Schooling			
No schooling/incomplete elementary school	1.452	34.3	32.2–36.4
Complete elementary school/incomplete high school	671	15.8	14.3–17.4
Complete high school/incomplete higher education	1.458	34.4	32.4–36.6
Complete higher education or higher	654	15.4	13.9–17.1
Race/skin color			
White	1.713	39.8	37.5–42.2
Black	331	7.2	6.3–8.2
Brown	2.117	51.4	49.2–53.7
Other	71	1.5	1.1–2.1
Marital status			
With spouse	2.312	59.7	57.9–61.6
Without spouse	1.921	40.3	38.4–42.1
Zone of residence			
Rural	512	8.1	7.2–9.1
Urban	3.721	91.9	90.9–92.8
Recommended consumption of vegetables and fruits			
No	1.750	42.4	40.2–44.7
Yes	2.483	57.6	55.3–59.8
Consumption of meat and/or chicken with excess fat			
No	2.702	63.0	60.9–65.1
Yes	1.531	37.0	34.9–39.1
Regular consumption of sweets			
No	3.343	78.5	76.8–80.1
Yes	890	21.5	19.9–23.2
Regular replacement of meals with snacks			
No	3.913	92.7	91.5–93.7
Yes	320	7.3	6.3–8.5
Regular consumption of soda and/or artificial juice			
No	3.256	76.5	74.7–78.2
Yes	977	23.5	21.8–25.3
Regular consumption of alcoholic beverages			
No	3.004	71.5	69.7–73.2
Yes	1.228	28.5	26.8-30.3
Abusive consumption of alcoholic beverages			
No	2.870	91.0	89.7–91.1
Yes	363	9.0	7.9–10.3
Smoking			
Never	3.221	76.3	74.4–78.1
Ex smoker	577	13.3	12.0–14.7
Current smoker	435	10.4	9.3–11.7
Physical inactivity			
No	1.986	46.6	44.6–48.7
Yes	2.247	53.4	51.3–55.4
Nutritional status			
Low weight	100	2.5	1.9–3.2
Eutrophic	1.564	40.1	38.3–41.9
Overweight	1.365	32.3	30.5–34.2
Obesity	1.047	25.1	23.5–26.9
Multimorbidity			
No	3.039	78.5	69.7–73.3
Yes	1.194	28.5	26.7–30.4

* Number of valid responses; 95% CI = 95% confidence interval.

**Table 2:** Bivariate analysis of factors associated with negative health self-assessment in women. Midwest Region, Brazil, National Health Survey, 2013.

Variables	Total (*n* = 4.233)	NSAH		PR	95% CI	*p* *
		*n*	% (95% CI)			
Age group (years)						
18–39	2.012	45	2.2 (1.6–3.1)	1.00		
40–59	1.478	108	7.8 (6.1–9.9)	3.56	2.32–5.46	<0.001
≥60	743	95	13.4 (10.4–17.1)	6.13	4.06–9.27	<0.001
Schooling						
No schooling/incomplete elementary school	1.489	173	12.4 (10.4–14.8)	7.78	3.80–15.90	<0.001
Complete elementary school/incomplete high school	651	24	3.7 (2.2–6.2)	2.32	0.96–5.62	0.062
Complete high school/incomplete higher education	1.405	39	2.6 (1.8–3.9)	1.65	0.74–3.69	0.218
Complete higher education or higher	688	12	1.6 (0.8–3.2)	1.00		
Race/skin color						
White	1.713	87	5.4 (4.1–7.0)	1.00		
Black	331	29	9.2 (6.1–13.8)	1.71	1.03–2.83	0.037
Brown	2.117	125	5.8 (4.6–7.3)	1.07	0.75–1.54	0.705
Other	71	7	14.0 (5.6–30.9)	2.59	1.04–6.48	0.042
Marital status						
With spouse	2.312	115	5.4 (4.3–6.7)	0.77	0.58–1.04	0.090
Without spouse	1.921	133	6.9 (5.6–8.5)	1.00		
Zone of residence						
Rural	512	31	6.6 (4.2–10.1)	1.10	0.69–1.77	0.665
Urban	3.721	217	5.9 (5.1–7.0)	1.00		
State of residence						
Federal District	1.067	66	5.8 (4.5–7.5)	1.0		
Goiás	1.385	78	5.9 (4.5–7.7)	1.02	0.70–1.48	0.938
Mato Grosso	795	48	6.4 (4.6–9.0)	1.11	0.73–1.70	0.631
Mato Grosso do Sul	986	56	6.0 (4.4–8.0)	1.03	0.70–1.54	0.887
Recommended consumption of vegetables and fruits						
No	1.750	131	8.0 (6.5–9.8)	1.00		
Yes	2.483	117	4.5 (3.6–5.7)	0.56	0.41–0.77	<0.001
Consumption of meat and/or chicken with excess fat						
No	2.702	167	6.0 (5.0–7.3)	1.00		
Yes	1.531	81	6.0 (4.6–7.7)	0.99	0.72–1.36	0.951
Regular consumption of sweets						
No	3.343	221	6.6 (5.7–7.8)	1.00		
Yes	890	27	3.7 (2.3–5.9)	0.56	0.34–0.91	0.019
Regular replacement of meals with snacks						
No	3.913	234	6.1 (5.2–7.1)	1.00		
Yes	320	14	5.3 (3.0–9.2)	0.87	0.48–1.57	0.641
Regular consumption of soda and/or artificial juice						
No	3.256	207	6.6 (5.6–7.8)	1.00		
Yes	977	41	3.9 (2.7–5.7)	0.59	0.39–0.89	0.013
Regular consumption of alcoholic beverages						
No	3.005	214	7.2 (6.1–8.4)	1.00		
Yes	1.228	34	3.1 (2.0–4.6)	0.43	0.27–0.66	<0.001
Abusive consumption of alcoholic beverages						
No	3.870	234	6.2 (5.3–7.2)	1.00		
Yes	363	14	4.1 (2.1–7.6)	0.65	0.34–1.27	0.211
Smoking						
Never	3.221	148	4.6 (3.7–5.7)	1.00		
Ex smoker	577	67	12.5 (9.4–16.5)	2.72	1.91–3.87	<0.001
Current smoker	435	33	7.8 (5.2–11.6)	1.70	1.08–2.68	0.022
Physical inactivity						
No	1.986	80	4.2 (3.2–5.4)	1.00		
Yes	2.247	168	7.6 (6.2–9.2)	1.81	1.30–2.51	<0.001
Multimorbidity						
No	3.039	67	2.6 (1.9–3.5)	1.00		
Yes	1.194	181	14.6 (12.3–17.2)	5.69	4.04–8.02	<0.001

NSAH = Negative Self-Assessment of Health; PR = Prevalence Ratio; 95% CI = 95% Confidence Interval; * Wald statistic.

**Table 3:** Multiple regression analysis of factors associated with negative health self-assessment in women. Midwest Region, Brazil, National Health Survey, 2013.

Variables	aPR	95% CI	SE	*p* *
Age group (years)				
18–39	1.00			
40–59	2.43	1.07–5.52	1.01	0.034
≥60	2.52	1.02–6.23	1.16	0.045
Schooling				
No schooling/incomplete elementary school	1.00			
Complete elementary school/incomplete high school	1.95	0.87–4.37	0.80	0.102
Complete high school/incomplete higher education	2.31	0.95–5.60	1.04	0.065
Complete higher education or higher	4.85	2.27–10.37	1.88	<0.001
Race/skin color				
White	1.00			
Black	1.59	0.96–2.63	0.41	0.068
Brown	1.02	0.71–1.47	0.19	0.893
Other	2.01	0.79–5.11	0.95	0.141
Marital status				
With spouse	1.00			
Without spouse	0.89	0.65–1.21	0.14	0.456
State of residence				
Federal District	1.00			
Goiás	0.71	0.49–1.03	0.13	0.071
Mato Grosso	0.79	0.52–1.20	0.17	0.275
Mato Grosso do Sul	0.74	0.50–1.09	0.15	0.126
Recommended consumption of vegetables and fruits				
No	1.00			
Yes	0.79	0.40–1.56	0.27	0.496
Regular consumption of sweets				
No	1.00			
Yes	0.75	0.45–1.25	0.19	0.270
Regular consumption of soda and/or artificial juice				
No	1.00			
Yes	0.79	0.52–1.20	0.17	0.266
Abusive consumption of alcoholic beverages				
No	1.00			
Yes	1.01	0.55–1.88	0.32	0.950
Smoking				
Never	1.00			
Ex smoker	1.30	0.90–1.89	0.25	0.160
Current smoker	1.22	0.79–1.88	0.27	0.370
Physical inactivity				
No	1.00			
Yes	1.55	1.11–2.15	0.26	0.009
Multimorbidity				
No	1.00			
Yes	6.93	3.47–13.85	2.44	<0.001

aPR = Adjusted Prevalence Ratio; 95% CI = 95%Confidence Interval; SE = Standard Error; * Wald Statistic. Note: Poisson multiple regression model adjusted by age group, education, race/skin color, marital status, state of residence, recommended consumption of fruits and/or vegetables, regular consumption of sweets, regular consumption of soft drinks, abusive consumption of alcoholic beverages, smoking, physical inactivity and multimorbidity.

## Data Availability

Available from: https://www.ibge.gov.br/estatisticas/sociais/justica-e-seguranca/29540-2013-pesquisa-nacional-de-saude.html, (accessed on 12 November 2021).
